# Metabolic Syndrome Triggered by High-Fructose Diet Favors Choroidal Neovascularization and Impairs Retinal Light Sensitivity in the Rat

**DOI:** 10.1371/journal.pone.0112450

**Published:** 2014-11-07

**Authors:** Magalie Thierry, Bruno Pasquis, Niyazi Acar, Stéphane Grégoire, Valérie Febvret, Bénédicte Buteau, Ségolène Gambert-Nicot, Alain M. Bron, Catherine P. Creuzot-Garcher, Lionel Bretillon

**Affiliations:** 1 INRA, UMR 1324 Centre des Sciences du Goût et de l'Alimentation, Eye and Nutrition Research Group, Dijon, France; 2 CNRS, UMR 6265 Centre des Sciences du Goût et de l'Alimentation, Dijon, France; 3 Université de Bourgogne, Centre des Sciences du Goût et de l'Alimentation, Dijon, France; 4 Department of Clinical Chemistry, University Hospital, Dijon, France; 5 Department of Ophthalmology, University Hospital, Dijon, France; Copenhagen University Hospital Roskilde and the University of Copenhagen, Denmark

## Abstract

Diabetic retinopathy and age-related macular degeneration are the leading causes of blindness in Western populations. Although it is a matter of controversy, large-scale population-based studies have reported increased prevalence of age-related macular degeneration in patients with diabetes or diabetic retinopathy. We hypothesized that metabolic syndrome, one of the major risk factors for type 2 diabetes, would represent a favorable environment for the development of choroidal neovascularization, the main complication of age-related macular degeneration. The fructose-fed rat was used as a model for metabolic syndrome in which choroidal neovascularization was induced by laser photocoagulation. Male Brown Norway rats were fed for 1, 3, and 6 months with a standard equilibrated chow diet or a 60%-rich fructose diet (*n* = 24 per time point). The animals expectedly developed significant body adiposity (+17%), liver steatosis at 3 and 6 months, hyperleptinemia at 1 and 3 months (two-fold increase) and hyperinsulinemia at 3 and 6 months (up to two-fold increase), but remained normoglycemic and normolipemic. The fructose-fed animals exhibited partial loss of rod sensitivity to light stimulus and reduced amplitude of oscillatory potentials at 6 months. Fructose-fed rats developed significantly more choroidal neovascularization at 14 and 21 days post-laser photocoagulation after 1 and 3 months of diet compared to animals fed the control diet. These results were consistent with infiltration/activation of phagocytic cells and up-regulation of pro-angiogenic gene expression such as *Vegf* and *Leptin* in the retina. Our data therefore suggested that metabolic syndrome would exacerbate the development of choroidal neovascularization in our experimental model.

## Introduction

Diabetic retinopathy (DR) and age-related macular degeneration (AMD) are the leading causes of visual loss in Western populations before and after the age of 50 years, respectively [1]. The association between DR and AMD remains controversial. Large-scale population-based studies have reported increased incidence or prevalence of AMD in patients with diabetes or DR [2], [3], [4], [5]. On the contrary, others have failed to find a similar relationship such as the Framingham Eye Study [6] and smaller case–control studies [7], [8]. Metabolic syndrome (MetS) is defined as a constellation of clinical criteria including visceral adiposity, elevated blood pressure, hypertriglyceridemia, insulin resistance, and elevated fasting glycemia. Its prevalence is high worldwide, although it varies from one country to another and depends on defining criteria. Using the NCEP (National Cholesterol Education Program) definition, MetS reached 14.1% of the population in France in 2006–2007 [9] and 22.9% in the US in 2010 [7]. MetS remains a major risk factor for the development of type 2 diabetes (T2D) [10]. The prevalence of diabetic retinopathy (DR) in the diabetic population is high: from 9.9% in T2D adults 18 years and older to 28.5% after the age of 40 years [1]. Diabetes plays a deleterious role on several structures in the eye including the cornea, the lens, the optical nerve head and the retina [11], [12]. Although no animal model of T2D recapitulates the late proliferative stages of DR [13], most studies on the consequences of diabetes on the retina were restricted to the vascular effects of hyperglycemia, including vasopermeability, endothelial cell proliferation and promoted neovascularization [14], [15]. However, far less is known on the adaptation and characterization of the retinal changes related to MetS. High-fructose diets have been associated with metabolic changes and excess weight gain that are typical of MetS and may predispose to T2D [14], [16], [17], [18]. This concern was supported mainly by observations in high-fructose-fed rodents that reported hyperinsulinemia, increase of body fat, and hepatomegaly associated with accumulation of lipids in the liver [19], [20]. The feeding of rats with high fructose is therefore a suitable and pertinent model for MetS [14]. Choroidal neovascularization (CNV) is the major complication of exudative AMD. The rupture of Bruch's membrane by laser impacts is a commonly used technique to induce CNV in animals [21]. In our study, we sought first to characterize the functional and gene expression changes in the retina of fructose-fed rats, and second to evaluate whether fructose-fed rats would be more prone to developing CNV than control animals.

## Materials and Methods

### 1. Ethical concerns

All procedures were conducted in accordance with the Association for Research in Vision and Ophthalmology Statements for the use of animals in ophthalmic and vision research and were approved by the local Animal Care and Use Committee (Comité d'Ethique de l'Expérimentation Animale nr 105, Dijon, France). Personal (nr 21CAE095) and institutional (nr B21231010EA) agreements were obtained according to French regulations.

### 2. Experimental diets

Standard and 60%-rich fructose diets (Table 1) were purchased from Sniff Spezialdiäten Gmbh (Soest, Germany).

**Table 1:** Composition of the experimental diets.

	Standard diet	Fructose-enriched diet
	In g per kg of diet	
Casein	180	180
Cornstarch	460	90
Sucrose	230	0
Fructose	0	600
Cellulose	20	20
Mineral mix (a)	50	50
Vitamin mix (b)	10	10
Fat: oil mix(c)	50	50

(a) Composition (g/kg): sucrose, 110.7; CaCO_3_, 240; K_2_HPO_4_, 215; CaHPO_4_, 215; MgSO_4_.7H_2_O, 100; NaCl, 60; MgO, 40; FeSO_4_.7H2O, 8; ZnSO_4_, 7H_2_O. 7; MnSO_4_.H_2_O, 2; CuSO_4_.5H_2_O, 1; Na_2_SiO_7_.3H_2_O, 0.5; AlK(SO_4_)_2_.12H_2_O, 0.2; K_2_CrO_4_, 0.15; NaF, 0.1; NiSO_4_.6H_2_O, 0.1; H_2_BO_3_, 0.1; CoSO_4_.7H_2_O, 0.05; KIO_3_, 0.04; (NH_4_)_6_Mo_7_O_24_.4H_2_O, 0.02; LiCl, 0.015; Na_2_SeO_3_, 0.015; NH_4_VO_3_, 0.01.

(b) Composition (g/kg): sucrose, 549.45; retinyl acetate, 1; cholecalciferol, 0.25; DL-tocopheryl acetate, 20; phylloquinone, 0.1; thiamine HCl, 1; riboflavin, 1; nicotinic acid, 5; calcium pantothenate, 2.5; pyridoxine HCl, 1; biotin, 1; folic acid, 0.2; cyanocobalamin, 2.5; choline HCl, 200; DL-methionine, 200; p-aminobenzoic acid, 5; inositol, 10.

(c) Composition of the oil mix (%): Rapeseed oil, 18. 7; Oleic oil, 38.1; Sunflower oil, 5; Palm oil, 38.1. Omega 6 to omega 3 ratio  = 7.5.

### 3. Animals

**Figure 1:**
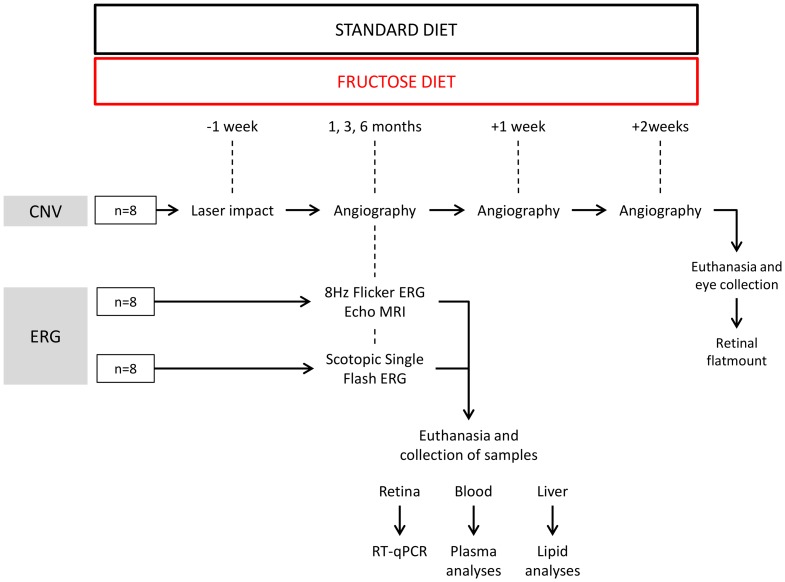
Flow chart of the experimental procedure. CNV: choroidal neovascularization, ERG: electroretinography.

Male Brown Norway rats (6 weeks of age, Charles River, L'Arbresle, France) were housed in controlled temperature (22±1°C) and humidity (55–60%) conditions with a 12-h light/12-h dark cycle (Animalerie Expérimentale, CSGA, Dijon, France). After a 7-day-long quarantine, the animals were randomly allocated to the experimental groups corresponding to the feeding of either of the two experimental diets during 1, 3 and 6 months (*n* = 24 per diet and per time point) (Figure 1). Rats had unrestricted access to food and deionized tap water.

### 4. Argon laser-induced choroidal neovascularization (CNV) in rats

One week before the end of each dietary period (1, 3 or 6 months) (Figure 1), the rats were anesthetized by intraperitoneal injection of ketamine (100 mg/kg, Imalgène 1000, Merial, Lyon, France) and xylazine (10 mg/kg, Rompun 2%, Bayer, Puteau, France). Pupils were dilated with 1% tropicamide (Mydriaticum, Laboratoires Théa, Clermont-Ferrand, France). Laser photocoagulation (532 nm, 300 mW, 50 ms, 75 µm, Vitra, Quantel Medical, Clermont-Ferrand, France) was unilaterally performed (5 to 7 spots per eye) in 8 animals per group. The laser spots were delivered around the optic nerve using a slit lamp delivery system and a glass coverslip as a contact lens. The validation of the injury was ascertained at the time of the laser shot by the appearance of a bubble.

### 5. Retinal and choroidal angiography

Retinal and choroidal angiography was performed every 7 days for the 3 weeks following CNV induction by confocal scanning laser ophthalmoscopy using Heidelberg Retinal Angiograph (HRA1, Heidelberg Engineering, Germany) (*n* = 8 rats per group) (Figure 1). Rats were anesthetized by intraperitoneal injection of ketamine (100 mg/kg, Imalgène 1000, Merial) and xylazine (10 mg/kg, Rompun 2%, Bayer). Pupils were dilated with 1% tropicamide (Mydriaticum, Laboratoires Théa). Fluorescein (0.15 mL of 1% saline solution per 300 g body weight, Sigma Aldrich, Saint Quentin Fallavier, France) and Indocyanine Green (ICG) (0.15 mL of 0.4% saline solution per 300 g body weight, Infracyanine, Serb, Paris, France) were injected intravenously via the penile vein. Single pictures and depth scan movies were taken between 5 and 10 min after dye administration. Photographs of the retinal and the choroidal vasculature were recorded at 488 nm for retinal vessel fluorescein angiography and at 795 nm for choroidal ICG angiography. Barrier filters at 500 and 810 nm provided the optimal cutoff at the respective peak fluorescence emission values for the two types of angiography. The size of the square scan field was set at 20° and 10°.

### 6. Semi-quantitative analysis of laser-induced CNV

CNV was semi-quantified in a double-blind manner by two independent investigators on images from ICG angiography 2 and 3 weeks after laser-induced CNV using ImageJ software. The CNV area was delimited on grey-scale images centered on the optic nerve head and measured in pixels. In the 256 values of the grey scale, the lower limit for the detection of CNV (i.e., hyperfluorescence) was 119±13 compared to 80±17 for background noise (mean ± SD, *n* = 8 independent determinations in different fundi). The ratio of the fluorescence of each laser impact to the optic nerve head area was calculated and averaged per eye (*n* = 5 impacts per eye, *n* = 8 rats per group).

### 7. Electroretinography

The electroretinograms (ERG) were recorded after 1, 3, and 6 months of feeding (*n* = 16 rats per group) (Figure 1), according to previously described procedures and ISCEV (International Society for Clinical Electrophysiology of Vision) guidelines [22]. Rats were dark-adapted overnight before the experiments. All further procedures were carried out under dim red light (λ<650 nm) at a constant temperature of 25°C. Rats were anesthetized by intramuscular injection of ketamine (100 mg/kg, Imalgène 1000, Merial) and xylazine (10 mg/kg, Rompun 2%, Bayer). Pupils were dilated with 1% tropicamide (Mydriaticum, Laboratoires Thea). After 10 min, rats were positioned on a warming plate, and the corneal electrodes were put in place. The ERG was recorded via corneal electrodes (thin gold wire with a 3-mm ring end) and reference and ground electrodes (silver needle) placed on the forehead and tail, respectively. The recording setup featured a Ganzfeld bowl, an amplifier, and a computer-based control and recording unit (RETI port/scan 21, Stasche & Finger GmbH, Roland Consult, Brandenburg, Germany). ERG responses were recorded from both eyes simultaneously after the rats were placed in the Ganzfeld bowl.

The first ERG examination corresponded to the scotopic single flash, scotopic threshold response (STR) and oscillatory potential (OP) recordings (*n* = 8 rats per group). The band-pass filter width was 1–300 Hz for the recording procedure of scotopic single-flash responses. The stimuli of the scotopic single-flash were recorded with ten increasing intensities from 0.0003 cd.s/m^2^ to 10 cds/m^2^. These responses were averaged with an inter-stimulus interval of 5 s (from 1 to 10 cd.s/m^2^) or 17 s (up to 0.3 cd.s/m^2^). The band-pass filter width was 0.2–30 Hz for the STR recording procedure. The STR stimuli were recorded with three increasing intensities: −4.60 log, −4.32 log, and −4.02 log cd.s/m^2^. A 2 s inter-stimulus interval was used between these stimuli. After amplification, the signal was digitized and processed. The amplitude and latency of the a- and b-waves were analyzed as previously described [23]. OPs were recorded as previously published [24]. The band-pass filter width was 200–500 Hz. Two stimuli were applied with an inter-stimuli interval of 17 s. After amplification, the signal was digitized and processed on the basis of means of amplitude and time-latency measurements of each of the four typically characterized peaks (OP1–OP4) [25]. The second flicker ERG examination consisted in fixed frequency 8.02 Hz light stimulation, in order to avoid subharmonic of the frequency of the power supply (set at 50 Hz) (*n* = 8 rats per group). The responses were recorded at ten increasing intensities from 0.0003 cd.s/m^2^ to 10 cd.s/m^2^ with an inter-stimulus interval of 0.1247 s.

### 8. Body composition analysis

As mentioned in Figure 1, whole body composition was analyzed by quantitative magnetic resonance imaging (EchoMRI 500, EchoMRI, Houston, Texas; Plateforme de phénotypage du petit animal, Université de Bourgogne, AgroSup Dijon, France). Animals were adapted to the environment of the EchoMRI analyzer at least 12 h before measurement with free access to food and water. Scans were taken by placing animals in a thin-walled plastic cylinder (3 mm thick, 6.8- or 8.2-cm inner diameter, based on body weight), with a cylindrical plastic insert added to limit the movement of the animals. Within the tube, the animals were briefly subjected to a low-intensity (0.05 Tesla) electromagnetic field to measure fat, lean mass, free water, and total body water, as described by Tinsley [26]. Briefly, this system generates a signal that modifies the spin patterns of hydrogen atoms within the subject, and uses an algorithm to evaluate the resulting T1 and T2 relaxation curves specific to each of the four components measured: fat mass, lean muscle mass equivalent, total body water, and free water. Quantitative magnetic resonance scans were taken in triplicate (*n* = 8 rats per group).

### 9. Collection of samples

The animals (*n* = 16 rats per group) were fasted overnight. The rats were deeply anesthetized with pentobarbital (Ceva, Santé animale, Libourne, France). Blood was collected from the abdominal artery in EDTA tubes and plasma was prepared by centrifugation (20 min, 3000 rpm) and stored at −80°C until further analysis. Rats were then euthanized by exsanguination. Retinas were dissected and stored in RNAlater solution (Qiagen, Courtaboeuf, France) until RNA extraction. The liver was collected and stored at −80°C until lipid and fatty acid analyses. Eyes were collected at the end of the CNV protocol for flatmounting of the retinas (see paragraph 2.13), two weeks after the end of the diet period (*n* = 8 per group) (Figure 1).

### 10. Lipid analyses

Lipids were extracted from the liver (*n* = 8 per group) according to the Folch method [27] and submitted to transmethylation of the fatty acids using boron trifluoride in methanol according to Morrison and Smith [28]. Fatty acid methyl esters were subsequently extracted with hexane and analyzed using gas chromatography on a Hewlett Packard Model 5890 gas chromatograph (Palo Alto, CA, USA) using a CPSIL-88 column (100 m, 0.25 mm i.d., 0.20-µm film thickness, Varian, Les Ulis, France) equipped with a flame ionization detector. Hydrogen was used as the carrier gas (inlet pressure, 210 kPa). The oven temperature was held at 60°C for 5 min, increased to 165°C at 15°C/min and held for 1 min and then to 225°C at 2°C/min and finally held at 225°C for 17 min. The injector and the detector were maintained at 250°C. Fatty acid methyl esters were identified by comparison with commercial and synthetic standards (Sigma Aldrich, L'Isle d'Abeau, France). The data were processed using the EZChrom Elite software (Agilent Technologies, Massy, France) and reported as a percentage of the total fatty acids.

The distribution of lipids into phospholipids, triacylglycerols, free fatty acids, free cholesterol, and cholesteryl esters in the neural retina (*n* = 8 per group) was determined using a combination of thin-layer chromatography on silica gel-coated quartz rods and flame ionization detection (Iatroscan system, Iatron, Tokyo, Japan), according to Ackman's technique [29] and published by our group [30]. The values obtained for each compound were corrected according to their response factor using specific calibration curves, as previously published [31]. Data were reported as a percentage of the total lipids in the sample.

### 11. Plasma analyses

Glycemia was evaluated using a blood glucose system (One Touch Ultra, Lifescan) (*n* = 8 rats per group). Cholesterol, triacylglycerol, HDL, LDL, and fructosamine were quantified by standard automatic analyzers at the Clinical Chemistry Department of the Dijon University Hospital (Dijon, France) (*n* = 8 rats per group). Plasma insulin and leptin levels (*n* = 8 rats per group) were quantified on a 96-well plate using the Rat Metabolic Magnetic Bead Panel and assessed by Luminex technology (Biorad Bioplex 200 system, Life Sciences, Marnes-la-Coquette, France). The analyses were performed according to the manufacturers' protocols (Milliplex kit, Merck Millipore).

### 12. Analysis of gene expression in the neurosensory retina

Total RNA from the neurosensory retina (*n* = 8 in each group) were extracted using a commercial kit following the manufacturer's procedure (RNAqueous, Applied Biosystems, Courtaboeuf, France). cDNA were prepared using oligo-dT as the primer and the High-Capacity RNA-to-cDNA Master Mix from Applied Biosystems. The quality and concentration of the cDNA samples were checked by qPCR analysis of glucuronidase beta (GUSB) expression using TaqMan technology (ABI7900 Fast Real-Time PCR system, Applied Biosystems). The expression of 61 genes (Table 2), including four control genes and genes coding for lipid metabolism, fatty acid transport, inflammation, neovascularization, and chemokines, was analyzed using qPCR with the TaqMan Array Plates FAST Custom (ABI7900 Fast Real-Time PCR system, Applied Biosystems).

**Table 2:** List of the genes which expression was analyzed by RT-qPCR.

Gene symbol	Gene name	Reference sequence
Abca1	ATP-binding cassette, subfamily A (ABC1), member 1	NM_178095.2
Abca4	ATP-binding cassette, subfamily A (ABC1), member 4	NM_001107721.1
Abcg1	ATP-binding cassette, subfamily G (WHITE), member 1	NM_053502.1
Akt2	v-akt murine thymoma viral oncogene homolog 2	NM_017093.1
Alox12	arachidonate 12-lipoxygenase	NM_001105798.1
Alox5	arachidonate 5-lipoxygenase	NM_012822.1
Angptl3	angiopoietin-like 3	NM_001025065.1
Apoa4	apolipoprotein A-IV	NM_012737.1
Apob	apolipoprotein B	NM_019287.2
Apoe	apolipoprotein E	NM_138828.3
Asmt	acetylserotonin O-methyltransferase	NM_144759.2
Ccl2	chemokine (C-C motif) ligand 2	NM_031530.1
Cd36	CD36 molecule (thrombospondin receptor)	NM_031561.2
Cyp27a1	cytochrome P450, family 27, subfamily a, polypeptide 1	NM_178847.2
Cyp46a1	cytochrome P450, family 46, subfamily a, polypeptide 1	NM_001108723.1
Cyp7a1	cytochrome P450, family 7, subfamily a, polypeptide 1	NM_012942.1
Edn1	endothelin 1	NM_012548.2
Fas	Fas (TNF receptor superfamily, member 6)	NM_139194.2
Foxa2	forkhead box A2	NM_012743.1
Gfap	glial fibrillary acidic protein	NM_017009.1
Hif1a	hypoxia-inducible factor 1, alpha subunit (basic helix-loop-helix transcription factor)	NM_024359.1
Hmgcr	3-hydroxy-3-methylglutaryl-Coenzyme A reductase	NM_013134.2
Htra1	HtrA serine peptidase 1	NM_031721.1
Igf1	insulin-like growth factor 1	NM_001082477.2
Il1b	interleukin 1 beta	NM_031512.2
Il6	interleukin 6	NM_012589.1
Insig1	insulin induced gene 1	NM_022392.1
Ipcef1	nteraction protein for cytohesin exchange factors 1	NM_001170799.1
Irs1	insulin receptor substrate 1	NM_012969.1
Itgb2	integrin, beta 2	NM_001037780.2
Lcat	lecithin cholesterol acyltransferase	NM_017024.2
Ldlr	low density lipoprotein receptor	NM_175762.2
Lep	leptin	NM_013076.3
Lepr	leptin receptor	NM_012596.1
Lpl	lipoprotein lipase	NM_012598.2
Lrp1	low density lipoprotein-related protein 1 (alpha-2-macroglobulin receptor)	NM_001130490.1
Mapk8	mitogen-activated protein kinase 8	NM_053829.1
Mtnr1a	melatonin receptor 1A	NM_053676.2
Mtnr1b	melatonin receptor 1B	NM_001100641.1
Mttp	microsomal triglyceride transfer protein	NM_001107727.1
Nos2	nitric oxide synthase 2, inducible	NM_012611.3
Nox1	NADPH oxidase 1	NM_053683.1
Nox3	NADPH oxidase 3	NM_001004216.1
Nr1d1	nuclear receptor subfamily 1, group D, member 1	NM_001113422.1
Nr1h3	nuclear receptor subfamily 1, group H, member 3	NM_031627.2
Pltp	phospholipid transfer protein	NM_001168543.1
Ppara	peroxisome proliferator activated receptor alpha	NM_013196.1
Ppard	peroxisome proliferator-activated receptor delta	NM_013141.2
Rela	v-rel reticuloendotheliosis viral oncogene homolog A (avian)	NM_199267.2
Rxra	retinoid X receptor alpha	NM_012805.2
Rxrb	retinoid X receptor beta	NM_206849.3
Rxrg	retinoid X receptor gamma	NM_031765.1
Slc2a2	solute carrier family 2 (facilitated glucose transporter), member 2	NM_012879.2
Slc2a4	solute carrier family 2 (facilitated glucose transporter), member 4	NM_012751.1
Srb1	scavenger receptor class B, member 1	NM_031541.1
Tnf	tumor necrosis factor (TNF superfamily, member 2)	NM_012675.3
Vegfa	vascular endothelial growth factor A	NM_001110333.1
Housekeeping genes		
Gadd45a	growth arrest and DNA-damage-inducible, alpha	NM_024127.2
Gusb	glucuronidase, beta	NM_017015.2
B2m	beta-2 microglobulin	NM_012512.2
18S	Ribosomal 18S sub-unit	X_03205

### 13. Quantification of phagocytic cells in flat-mounted retinas

Rats fed 3 months with standard or fructose diets and submitted to laser-induced CNV were euthanized and enucleated 3 weeks after laser photocoagulation (Figure 1). The eyeballs were fixed in 4% paraformaldehyde (Sigma-Aldrich, Saint Quentin Fallavier, France) for 50 min, washed in PBS azide and stored at 4°C in PBS until preparation of retinal flatmounts. The cornea was incised, the lens was taken out, and four radial cuts were made on the eyecups. The vitreous was removed with forceps, and the retinas were gently isolated and flattened on microscope slides and circled by a hydrophobic pen (Dakopen, Dakocytomation, Trappes, France) in 200 µL of a blocking solution (1% BSA in PBS pH 6.8, 0.5% Tween 20) for 1 day and stored at 4°C under stirring. PBLec washing solution (PBS1X, pH 6.8, 0.1 mM Cacl_2_, 0.1 mM MgCl_2_, 1% Tween 20) was used twice to wash the retinal flatmounts (200 µL per washing for 5 min). Primary mouse antibody raised against rat anti-CD68 (dilution 1∶100) (MCA341R, AbD Serotec) was incubated overnight at 4°C under stirring. Five washings with PBS1X were then carried out, and retinal flatmounts were incubated with secondary antibody (dilution 1∶100) (A1101, anti-mouse Alexa 488, Life Technologies) for 2 h. Five washings in PBS1X were performed and the slides were coverslipped with a fluorescence mounting medium (Dakocytomation, Trappes, France). Retinas were examined under a confocal fluorescence microscope (Leica SP2, Leica Microsystems, Wetzlar, Germany, Plate-forme DIMACELL, Dijon, France). Images (165 µm×165 µm) were taken for quantification of the fluorescence in the experimental and fellow retinas using ImageJ software. Images from confocal fluorescence microscopy were converted in binary mode. The number of pixels corresponding to the value of 255 on the gray scale was considered as CD68-positive cells. Five images per eye for each diet (standard and fructose diets in both experimental and fellow retinas) were evaluated and averaged.

### 14. Statistical analyses

All analyses were conducted using GraphPad Prism software version 4.0. The level of statistical significance of the two-tailed tests was set at *p*≤0.05 and 0.01. A nonparametric Mann & Whitney test was used to compare body fat, circulating insulin and leptin levels, fatty acids, and lipid classes in the liver and non parametric Kruskal-Wallis test for quantification of CNV and CD68-immunostaining in the standard and fructose groups. Statistical analysis of gene expression was performed using integrated statistical analysis of data assist software (Student *t*-test) and a heat map was performed using the fold change values. Fold changes above the value of 1.5 was considered as significant. Dark green was used to represent a 10-fold change downregulation of gene expression, dark red was used to represent 7-fold change upregulation of gene expression. The levels of statistical significance were set at *p*≤0.1, 0.05, and 0.01.

## Results

### 1. Fructose diet triggered metabolic syndrome in the rat

**Figure 2:**
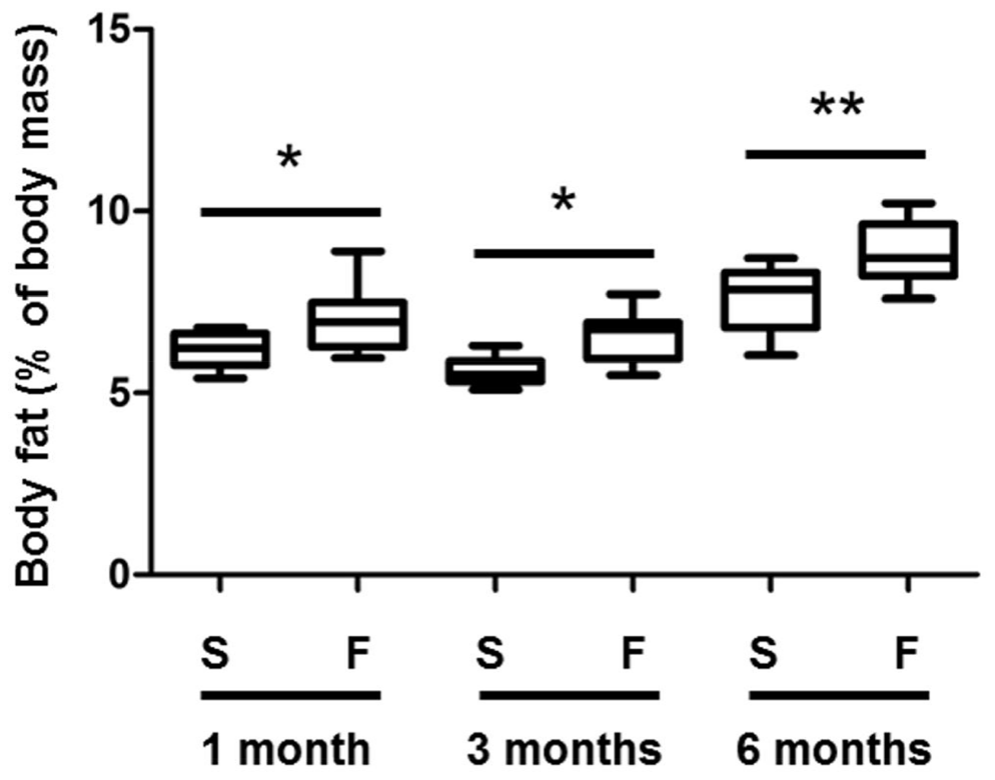
Body fat is increased in rats fed with a 60%-rich fructose diet. Body fat (expressed as % of body mass) was quantified by EchoMRI in the rat after 1, 3, and 6 months of feeding with either the standard (S) or 60%-rich fructose (F) diet. * and ** statistically different at *p*≤0.05 and 0.01, respectively (Mann & Whitney test). The bottom and top of the box are the first and third quartiles, and the band inside the box is the median. The ends of the whiskers are 1 standard deviation above and below the mean of the data (*n* = 8 rats per group).

**Figure 3:**
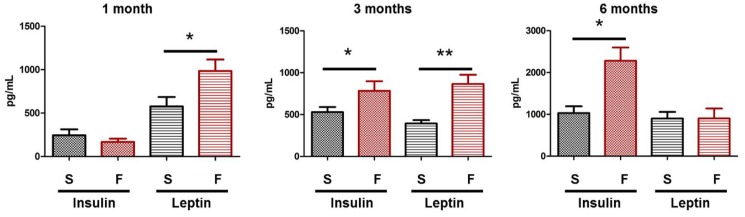
Insulinemia and leptinemia are increased in rats fed with a 60%-rich fructose diet. Plasma insulin and leptin (expressed in pg per mL of plasma) were quantified in the rat after 1, 3, and 6 months of feeding with either the standard (S) or 60%-rich fructose (F) diet. * and ** statistically different at *p*≤0.05 and 0.01, respectively (Mann & Whitney test). Values are given as means ± SD (*n* = 8 rats per group).

**Figure 4:**
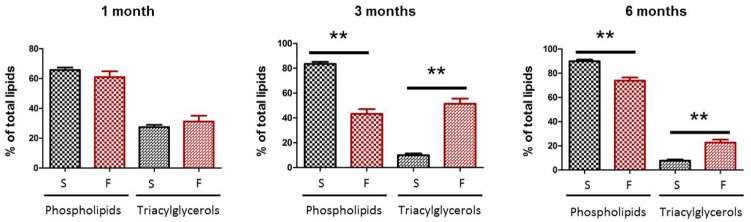
Liver steatosis is increased in rats fed with a 60%-rich fructose diet. Liver steatosis was evaluated by quantifying triacylglycerols (expressed in % of total lipids) in the liver of the rat after 1, 3, and 6 months of feeding with either the standard (S) or 60%-rich fructose (F) diet. ** statistically different at *p*≤0.01 (Mann & Whitney test). Values are given as means ± SD (*n* = 8 rats per group).

The fructose diet did not affect body weight (data not shown), but significantly increased body fat by 17% compared to rats of the control group at the corresponding ages (Figure 2). Plasma analyses revealed significant hyperleptinemia after 1 and 3 months of fructose feeding (*p* = 0.03 and 0.001, respectively), and hyperinsulinemia at 3 and 6 months (*p* = 0.05 and 0.04, respectively) (Figure 3). On the other hand, glycemia and plasma levels of HDL and LDL cholesterol and fructosamine remained constant (data not shown). The analysis of the lipid classes in the liver revealed a significant increase of triacylglycerol levels after 3 and 6 months of diet (five- and threefold increase, respectively, Figure 4), highlighting the development of liver steatosis induced by the fructose diet. As expected, increased liver triacylglycerols were associated with accumulation of palmitic and oleic acids (Table 3), which remain the prominent fatty acids accumulating at the time of steatosis [32], [33].

**Table 3:** Fatty acid composition of total lipids in the liver of rats fed with either the standard or 60%-rich fructose diet during 1, 3, and 6 months.

		Groups					
		1 month		3 months		6 months	
		Standard	Fructose	Standard	Fructose	Standard	Fructose
Fatty acids	C16:0 (palmitic acid)	971±93.7	1218±210.1 **	1116±39.0	1790±213.9 **	1081±179.7	1234±105.0
	C18:0 (stearic acid)	651±39.5	649±98.0 *	983±52.4	392±108.3 **	857±152.4	732±89.4 **
	C18:1 n-9 (oleic acid)	894±154.8	1220±335.8	858±126.4	1795±63.6 **	1103±168.3	1560±253.4
	C18:2 n-6 (linoleic acid)	579±76.6	607±80.2	586±36.8	280±67.2 **	587±101.6	487±45.3
	C22:6 n-3 (docosahexaenoic acid)	241±17.0	260±36.0	275±26.3	95±35.5 **	290±38.6	246±34.2

* and **, statistically different from the data in the corresponding standard group at *p*≤0.05 and 0.01, respectively (Mann & Whitney test). Values are means ± SD (n = 8 per group).

### 2. Metabolic syndrome favored laser-induced choroidal neovascularization in the rat via activation/infiltration of phagocytic cells in the retina

**Figure 5:**
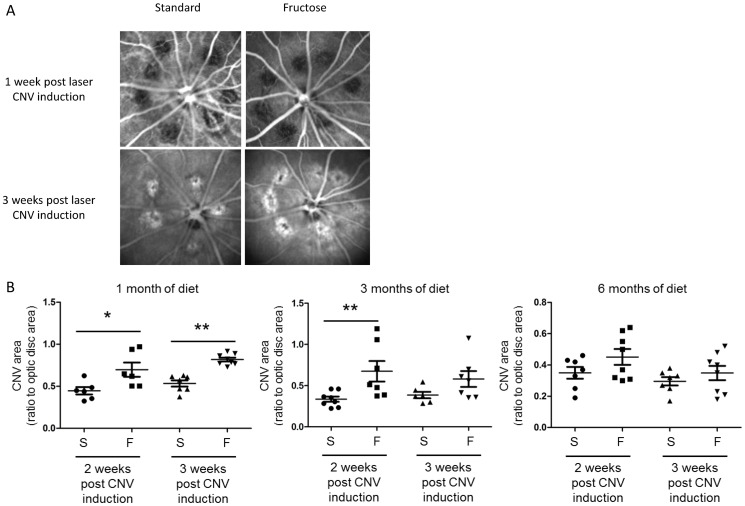
Laser-induced choroidal neovascularization (CNV) is enhanced in rats fed with a 60%-rich fructose diet. **A**. Representative images of choroidal indocyanine green angiography taken after 1 and 3 weeks post laser-induced CNV in rats fed with either the standard or 60%-rich fructose diet. Images were taken at 20° by confocal scanning laser ophthalmoscopy at 795 nm. The black holes in the eye fundi at 1 week post laser-induced CNV correspond to the breaks created by the laser spots (532 nm, 300 mW, 50 ms, 75 µm) in the retinal pigment epithelium and Bruch's membrane. CNV correspond to the filling of the new vessels by indocyanine green. **B**. Semi-quantification of CNV (ratio between the area of indocyanine green fluorescence and optic disc area) at 2 and 3 weeks after laser-induced CNV in the rat fed during 1, 3, and 6 months with either the standard (S) or 60%-rich fructose (F) diet. * and ** statistically different at *p*≤0.05 and 0.01, respectively (Kruskal-Wallis test). Values are given as individual data and means ± SD (*n* = 8 rats per group).

**Figure 6:**
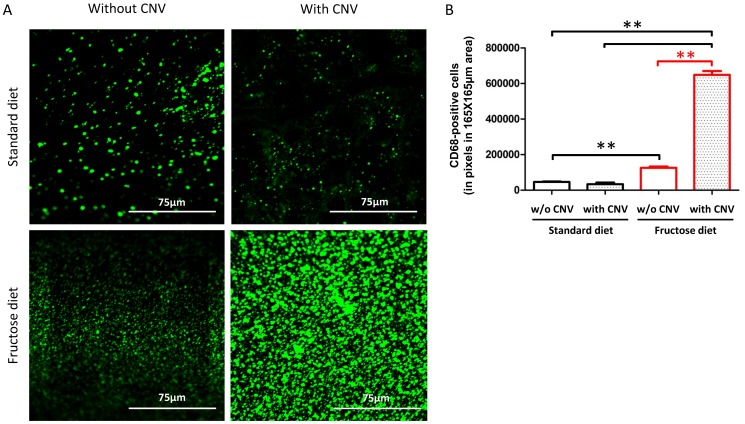
The number of CD68-positive cells is increased in the retina of rats fed with a 60%-rich fructose diet and submitted to laser-induced choroidal neovascularization (CNV). **A**. Representative confocal images of CD68-positive cells (revealed by an Alexa 488-labelled secondary antibody) in flat-mounted retinas of rats fed during 3 months with either the standard or the 60%-rich fructose diet and submitted or not to laser-induced CNV. Images corresponding to 165 µm×165 µm of the retinal area were taken 3 weeks post laser-induced CNV. **B**. Quantification of CD68-positive cells in flat-mounted retinas of rats fed during 3 months with either the standard or fructose diet, and submitted or not to laser-induced CNV. ** statistically different at *p*≤0.01 (Kruskal-Wallis test). Values are expressed in pixels of fluorescence in the area corresponding to 165 µm×165 µm of the retina; values are given as means ± SD.

CNV did not develop 1 week after laser, as illustrated by the lack of fluorescence in the scars created by the laser spots (Figure 5A). Hyperfluorescent areas appeared in the lesions compared to the surrounding choroid after 2 (images not shown) and 3 weeks (Figure 5A). These areas correspond to filling of the vessels with ICG dye and thus to the neovascularization process. Figure 5B presents semi-quantitative CNV data. A significant CNV enhancement was observed after 1 month of fructose-feeding 2 and 3 weeks after laser injury compared to the standard diet (*p* = 0.03, *p* = 0.0003, respectively). After 3 months of feeding, fructose-fed rats showed exacerbated CNV development only at week 2 after laser impacts (*p* = 0.0065). No significant CNV was observed after 6 months of feeding with the high-fructose diet (*p*>0.05). Analysis of CD68-positive immunostaining in flat-mounted retinas (Figure 6A) showed a significant increase of CD68 expression in fructose-fed rats compared to standard-fed animals in fellow retinas (*p*≤0.01, Figure 6B). CNV significantly enhanced CD68 immunostaining in fructose-fed rats, suggesting massive infiltration of circulating macrophages and/or activation of resident microglia.

### 3. Fructose diet-modulated gene expression in the retina

**Figure 7:**
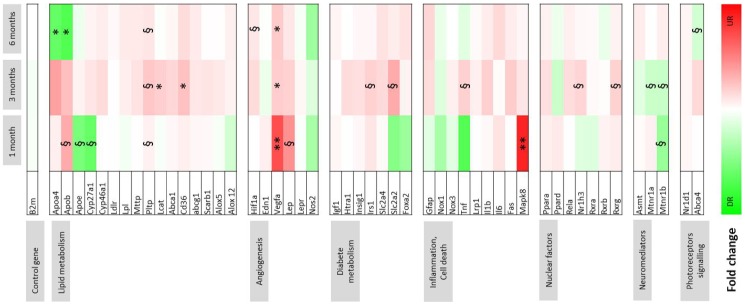
Gene expression changes in the neurosensory retina induced by a 60%-rich fructose diet in the rat. Data of gene expression were obtained by RT-qPCR and represented as a heat map. Fold change is indicated in the color scale, green indicates downregulation (up to 10-fold repression) and red indicates upregulation (up to 7-fold induction) in the retina of rats fed with the 60%-rich fructose diet by comparison to the rats fed with the standard diet at the corresponding ages (*n* = 8 per group). §, * and **, statistically different at *p*≤0.1, 0.05 and 0.01, respectively (Student *t*-test, *n* = 8 per group).

Figure 7 presents the data of gene expression in the retina of fructose-fed rats by comparison to animals fed the standard diet. The results show significant upregulation of the proangiogenic genes *Vegfa* at 1 month (*p*≤0.01), 3 months (*p*≤0.05), and 6 months (*p*≤0.05) and *Hif1a* at 6 months (*p*≤0.1). Meanwhile *Leptin* (*p*≤0.1) and *Mapk8* genes (*p*≤0.01) were upregulated at 1 month. In contrast, melatonin receptor genes (*Mtnr1a* and *Mtnr1b*) were downregulated after 1 and 3 months of diet (*p*≤0.1). At 3 months, fructose feeding induced upregulation of several genes coding nuclear factors (*Rxrg*, *Nr1h3*, *p*≤0.1), proteins involved in lipid metabolism including *Pltp* (p≤0.1), *Lcat*, and *Cd36* (p≤0.05), cell death inflammation (*Tnf*, *p*≤0.1), and diabetes (*Irs1*, *Slc2a2*, *p*≤0.1). After 6 months of fructose feeding, we noted down regulation of genes involved in photoreceptor signaling (*Abca4*, *p*≤0.1) and lipid metabolism: *Apoa4* and *ApoB* (*p*≤0.05), although *Pltp* remained upregulated (*p*≤0.1).

### 4. Rod sensitivity to light was partially lost in metabolic syndrome

**Figure 8:**
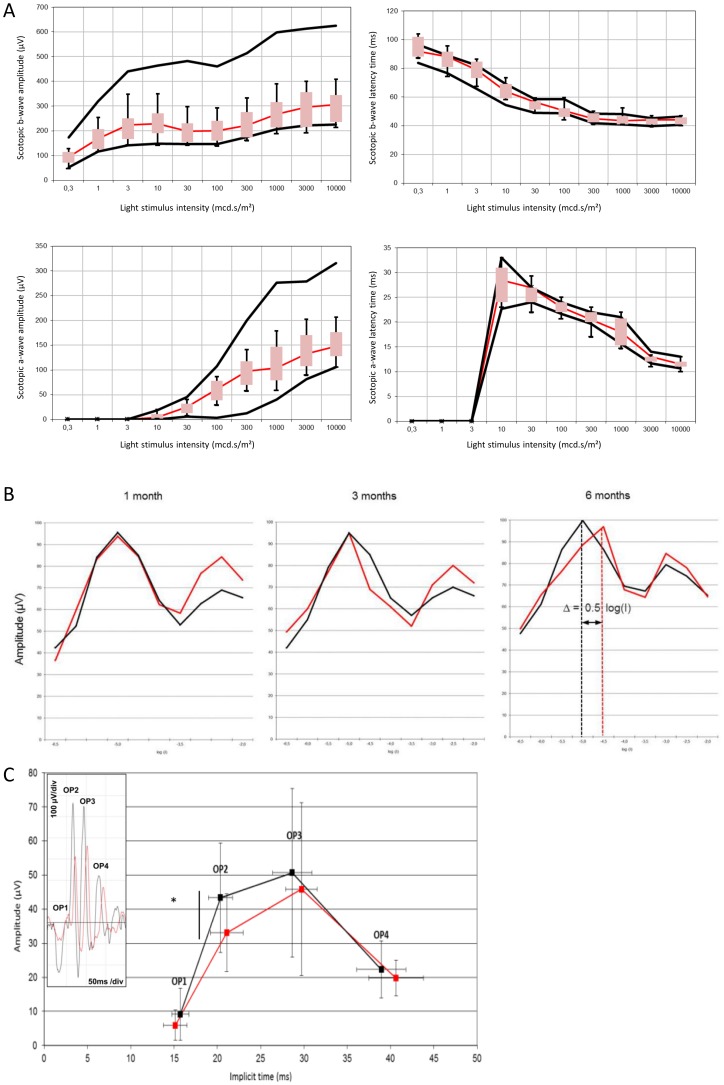
Electroretinographic changes induced by a 60%-rich fructose diet in the rat. **A**. Scotopic single flash response of dark-adapted rats after 6 months feeding with either the standard or 60%-rich fructose diet (*n* = 8 per group). Scotopic b-wave and a-wave amplitudes (in µV), b-wave and a-wave latency times (in ms) were plotted as a function of light stimulus intensity (in mcd.s/m^2^). Black lines show normal range given by the 5% and 95% percentile of the rats fed with the standard diet (*n* = 8 per group). Box plots correspond to the 5% and 95% percentile of the rats fed with the 60%-rich fructose diet (*n* = 8 per group). Statistically significant changes are observed when box plots are outside of the range given by the 5% and 95% percentile of the rats fed with the standard diet. **B**. 8.02 Hz Flicker electroretinographic data of the rats fed with either the standard (black traces) or 60%-rich fructose (red traces) diet during 1, 3, and 6 months. Data of the amplitude are plotted in amplitude (in µV) of the electroretinographic response as a function of light stimulus intensity (log value) (*n* = 8 per group). The first peak corresponds to the maximal response of rods and the second peak to the maximal response of cones [34]. A shift to the right of the first peak in the 60%-rich fructose fed rats is indicative of the loss of sensitivity of the rod photoreceptors. **C**. Amplitude (in µV) and implicit time (in ms) of the four oscillatory potentials (OP) of dark-adapted rats fed during 6 months with either the standard (black traces) or 60%-rich fructose (red traces) diet. Representative raw ERG is presented in the insert to illustrate the reduced amplitude of OP2, and to a lesser extent of OP3, in rats fed a 60%-rich fructose diet (in red) compared to standard diet (in black). * statistically different at *p*≤0.05 (Mann & Whitney test). Values are given as means ± SD (*n* = 8 per group).

The amplitudes and implicit times of the a- and b-waves of the ERG are shown in Figure 8A. No significant effect of fructose feeding was reported, although a trend was observed for the reduction of the b-wave amplitude in fructose-fed rats. To further characterize the changes induced by the fructose diet, additional protocols were performed. Flicker ERG is a suitable technique to specifically record rod and cone sensitivity to light stimulus [34]. It was recorded after 1, 3, and 6 months in both fructose-fed rats and animals fed the standard diet. The results are shown in Figure 8B. The data demonstrated no effect of fructose on rod or cone sensitivity at 1 and 3 months. On the contrary, the data obtained in rats fed with fructose for 6 months exhibited a shift to the right of the maximal response of rods (Δ = 0.5 log(I)). This illustrates that a higher light intensity was required to reach the maximal response of rods, suggesting partial loss of rod sensitivity induced by the fructose diet. Oscillatory potentials (OPs) were further analyzed. The amplitudes and implicit times of the four OPs are presented in Figure 8C. A significant decrease of OP2 was observed in fructose-fed rats as illustrated in the insert of Figure 8C (*p*≤0.05).

## Discussion

MetS is a major risk factor for T2D and DR. Most studies on DR focused on the mechanisms and consequences of hyperglycemia in retinal vessels. These data highlighted the prominent role of oxidative stress, inflammation and hemostatic factors including VEGF secretion in the complications of DR, namely retinal edema and retinal neovascularization [12], [35], [36]. Our data showed that feeding Brown Norway rats for 1 and 3 months with a 60%-rich fructose diet induced body adiposity and hyperleptinemia. Hyperinsulinemia was reported after 3 and 6 months of diet (Figure 3), suggesting the development of insulin resistance in a prediabetic state [37]. These data are consistent with previous reports using fructose diet as a model to induce insulin resistance in rats [38], [39] and more generally describing some features of MetS [40].

Most large-scale population-based studies reported increased incidence or prevalence of AMD in patients with diabetes or DR [2], [3], [4], [5], with the exception of the Framingham Eye Study [6] or smaller case–control studies [7], [8]. The matter is subject to controversy. At least two studies reported a consistent and similar association between diabetes and neovascular AMD, with an odds ratio of 1.88 (95% CI, 1.07–3.31) in the Age-Related Eye Disease Study [2] and 1.81 (95% CI, 1.10–2.98) in the EUREYE study [5]. Others found an association with geographic atrophy [4]. Nevertheless and interestingly, in those reports diabetes and early AMD appeared to be unrelated. These data reinforced our hypothesis that MetS as a risk factor for T2D is a promoting factor for neovascular complications in the retina.

On the one hand, the primary objective of this study was to characterize the adaptation of the retina to MetS. Flicker ERG data revealed partial loss of rod sensitivity to light stimulus after 6 months of fructose feeding (Figure 8B). This is consistent with downregulation of the *Abca4* gene after 6 months of fructose feeding, accounting for ABCA4 being physiologically involved in the retinoid cycle and mutations in *Abca4* being associated with cone and rod dystrophies [41]. It was previously reported that the sensitivity of photoreceptors was affected in DR and could be attributed to transduction abnormalities [42]. Alterations of photoreceptors were consistently observed in a model of streptozotocin-induced diabetic rats [43]. We also suggested a slight decrease of functionality of inner retinal cells by scotopic standard ERG, since the b-wave amplitude was lowered, although the difference from controls failed to reach statistical significance. We observed a decrease of OP2 amplitude in fructose-fed rats. Although the exact origin of OPs is still uncertain, OPs most probably reflect neuronal activity of bipolar cells and amacrine cells [44]. Several studies pointed out the association between alterations of OPs and diabetes. Indeed OP2 and OP4 responses were delayed in streptozotocin-induced diabetic rats [45]. Previous studies reported the association between the reduction of OP amplitudes and the severity of diabetic retinopathy [46], [47], [48], or even with the greater probability of developing DR [49]. Furthermore, patients with retinopathy and cone-rod diseases presented delayed OP2 despite normal b-wave peak times [50].

On the other hand, the secondary aim of our study was to investigate whether MetS represents a favorable environment for the development of CNV. Laser photocoagulation was used to trigger neovascularization, as used by others [21]. Laser spots create damage in the Bruch membrane that favors endothelial cells, pericytes, and inflammatory cells entering the RPE and sub-retinal space and forming CNV [21]. The present data showed that the consequences of a fructose diet were associated with exacerbated development of CNV after 1 and 3 months of feeding. We observed massive infiltration of CD68-positive phagocytic cells in the retina of rats submitted to laser injury and fed with fructose compared to rats fed with the standard diet. These cells may either be infiltrating macrophages and/or activated microglia. Enhanced CNV was correlated with overexpression of *VegfA* and *Leptin* genes in the retina. Leptin was shown to promote angiogenesis [51] and retinal neovascularization [52]. Various growth factors, including VEGF, are involved in the development of neovascularization [36]. Our data consistently illustrated the association between VEGF induction and enhanced CNV at early phases of MetS, i.e., at 1 and 3 months. In our model, MAPK8 was upregulated at 1 month. MAPK8 is a member of MAPK family that encompasses Jun kinases (JNK). JNKs are critical factors in the development of CNV, especially via the activation of macrophages [53]. Using the same model of CNV development as ours in mice, Du and coworkers elegantly showed that JNK is required for macrophage recruitment and demonstrated that JNKs inhibition and JNK1 knock-down reduced VEGF expression and lowered CNV [54]. In our model, MAPK8 overexpression was transient and restricted to 1 month, but might be sufficient to promote macrophage infiltration in the retina and further induce VEGF expression over a longer period of time, up to 6 months. The cholesterol-27-hydroxylase (CYP27A1) gene was significantly downregulated at 1 month in fructose-fed rats. The association between CYP27A1 and enhanced development of CNV in fructose-fed rats is consistent with data in CYP27A1 knock-out animals, showing vascular changes in the retina, including sprouting of vessels from the choroid into the neurosensory retina [55].

It must be noted that these changes in gene expression were observed in retinas of fructose-fed animals that were not submitted to CNV induction. Our findings therefore strongly support the notion that MetS constitutes a favorable environment for the development of CNV. In this context, the aforementioned effects cannot be associated with the well-recognized role of hyperglycemia in DR, since the fructose diet did not induce elevated glycemia. Our work therefore adds a new paradigm for the development of CNV in the context of MetS. We suggest that MetS per se may trigger mechanisms that are required for the development of neovascularization, including gene expression changes (*Mapk*, *Vegf*, and *Leptin* induction) and macrophage infiltration. Both circulating and *in situ* leptin and VEGF may be the key factors for the development of CNV in MetS, given that elevated leptin levels were reported to increase the risk for MetS in humans [56]. In the present study, elevated plasma leptin levels were associated with enhanced CNV at 1 and 3 months, whereas similar to controls hyperleptinemia was reported at 6 months while no CNV was promoted. Secondly, in a large clinical study investigating 1802 patients, elevated circulating VEGF levels were positively associated with MetS [57].

We must acknowledge several limitations in our study. First, feeding rats with fructose remains a model of MetS. Visceral adiposity, elevated blood pressure, hypertriglyceridemia, insulin resistance, and elevated fasting glycemia are hallmarks of MetS in humans. Accounting the lifespan of rats compared to humans, our results in rats 7 weeks of aged fed from 1 to 6 months may be relevant to MetS in young to middle-aged adults. We reported increased body fat, hyperinsulinemia, hyperleptinemia, but no hypertriglyceridemia, as was previously shown in rats fed with fructose [58]. Therefore, dietary exposure to fructose would merely be considered as a partial model of MetS, by predisposing cells and organs to chronic conditions of MetS [14].

This work showed, in a timely manner, the limitation of the time-course consequences of fructose feeding since no hyperleptinemia was detected after 6 months. This remediation of the effects may likely be due to the organism's long-term adaptive response. Nevertheless, one should note the consistency of these results given that leptin was considered a pro-angiogenic factor and no increased CNV was observed at this time point. Fructose diets are reported to promote oxidative stress and inflammation [14]. Although we did not ascertain these consequences in the retina, our data showing macrophage infiltration and/or microglia activation in the retina of fructose-fed rats is consistent with this hypothesis. As recently published, macrophages may be activated by oxidative damage in the retina and may participate in AMD pathogenesis [59]. Secondly, laser-induced neovascularization must be considered a model for CNV, but not for AMD. Until now, no animal model shows all the features of AMD, especially because no rodent model has a macula and spontaneously develops CNV. Despite its artificial nature, the laser model is currently the standard animal model of CNV and is widely used in both fundamental research and preclinical trials [21].

In conclusion, this study clearly showed that feeding rats with a high-fructose diet has detrimental consequences on the sensitivity of rod photoreceptors to light. In addition, high fructose generates a favorable environment for the development of neovascularization in the retina.
